# Investigation of chronic musculoskeletal pain (third report): with special reference to the importance of neuropathic pain and psychogenic pain

**DOI:** 10.1007/s00776-014-0567-6

**Published:** 2014-04-16

**Authors:** Masaya Nakamura, Yuji Nishiwaki, Masahiko Sumitani, Takahiro Ushida, Toshihiko Yamashita, Shinichi Konno, Toshihiko Taguchi, Yoshiaki Toyama

**Affiliations:** 1 Department of Orthopaedic Surgery, School of Medicine, Keio University, 35 Shinanomachi, Shinjuku, Tokyo, 160-8582 Japan; 2 Department of Environmental and Occupational Health, School of Medicine, Toho University, Tokyo, Japan; 3 Department of Anesthesiology and Pain Relief Center, The University of Tokyo Hospital, Tokyo, Japan; 4 Multidisciplinary Pain Center, Aichi Medical University, Aichi, Japan; 5 Department of Orthopaedic Surgery, Sapporo Medical College, Sapporo, Japan; 6 Department of Orthopedic Surgery, Fukushima Medical University School of Medicine, Fukushima, Japan; 7 Department of Orthopaedic Surgery, Yamaguchi University, Yamaguchi, Japan

## Abstract

**Background:**

The previous epidemiological surveys conducted in Japan revealed that once the vicious cycle of chronic musculoskeletal pain begins, it is difficult to disrupt the cycle. This finding suggests the existence of problems with the conventional approaches to treatment of chronic musculoskeletal pain. The purpose of this study was to investigate the characteristics of patients with chronic musculoskeletal pain focusing on neuropathic and psychogenic pain.

**Methods:**

The questionnaire was sent again to the 660 subjects found to have persistent chronic pain in the epidemiological surveys conducted in 2011. Responses were collected from 588 subjects (response rate 90 %).

**Results:**

Of the 588 responders, 365 (62 %) complained of persistent chronic pain. Among them, 128 (35 %) were still receiving treatment and 193 (53 %) had discontinued treatment. The degree of satisfaction with the treatment was low, and 66 % of the patients had switched the medical facility that they visited to receive treatment. The cited reasons for the change in the medical facility visited and discontinuation of treatment were “treatment was ineffective,” “I did not have sufficient time,” “I thought I could take care of it myself,” and “Treatment seemed to be unnecessary”. Involvement of neuropathic pain was suggested in 20 % of all the patients with chronic pain. As the PainDETECT Score rose, the Visual Analog Scale (VAS) score became higher and the change of medical facility for treatment also increased. The Pain Catastrophizing Scale score was correlated positively with the VAS score. The Hospital Anxiety and Depression Scale score was significantly correlated with the VAS score and the duration of pain.

**Discussion:**

The results of this survey indicated that the chronic course of musculoskeletal pain may be attributable to the following factors: (1) lack of appropriate treatment of neuropathic pain and psychogenic pain, and (2) insufficient awareness/knowledge among patients about chronic musculoskeletal pain.

## Introduction

The National Livelihood Survey provides data on symptoms currently prevalent in the Japanese general population. According to this survey, low back pain, shoulder stiffness, joint pain and other types of pain are highly ranked [1]. However, while attempting to devise countermeasures against chronic pain among Japanese people, we faced shortage of even basic information concerning the types of pain. Taking this background into account, we initiated the “longitudinal investigation of chronic musculoskeletal pain” in 2010. Until date, we have reported, based on the results of this survey, the prevalence of chronic musculoskeletal pain (15.4 %), the frequency of new onset of this type of pain (11.1 %), and the risk factors for the onset of chronic pain in the Japanese population. The investigation additionally revealed that chronic pain was frequently persistent (45.2 %), and that the risk factors for persistent pain were a VAS score of ≥7, duration of pain of ≥5 years, and pain affecting the lower back. Of the responders complaining of persistent chronic pain, more than 80 % had a history of treatment; about 30 % were still receiving treatment at the time of the investigation, while 50 % had discontinued treatment because of poor satisfaction with the outcome of treatment [2, 3]. These findings suggest that once the vicious cycle of chronic musculoskeletal pain begins, it is difficult to disrupt the cycle, and that the conventional approaches to treatment of chronic musculoskeletal pain may involve problems. The present survey was undertaken in the same subjects as those in the previously performed mail-based survey to characterize them with a chronic course of musculoskeletal pain, with emphasis laid on the possible involvement of neuropathic pain or psychogenic pain, and identification of problems with the conventional approaches to treatment.

## Methods

The questionnaire was mailed to 660 subjects who complained of persistent chronic pain in both the epidemiological surveys of 2010 and 2011 according to the mail-based survey panel maintained by Nippon Research Center, Ltd. [2, 3]. Responses were collected from 588 subjects (response rate, 90 %). The questionnaire used in this survey contained questions to determine information on the basic demographic characteristics of the subjects (gender, age, location of living, occupation, etc.), information about the chronic musculoskeletal pain (severity, location, duration, presence/absence of treatment, treating medical facility, therapeutic regimen used, treatment period, efficacy, degree of satisfaction with treatment), and information about neuropathic pain (PainDETECT score) [4] or psychogenic pain (Hospital Anxiety and Depression scale: HADS, Pain Catastrophizing Scale: PCS) [5, 6]. The subjects were divided into three categories according to the PainDETECT scores: the Non-neuropathic pain (NP) group (score of 12 or less; low likelihood of involvement of neuropathic pain), the Suspected NP group (score of 13–18; possible involvement of neuropathic pain), and the NP group (score of 19 or higher; strong suggestion of the involvement of neuropathic pain). The HADS consisted of HADS-anxiety (7 anxiety-related items: HADS-A) and HADS-depression (7 depression-related items: HADS-D). The responders were divided according to the HADS-A and HADS-D scores (21 at the maximum each) into 3 categories: score of 7 or less (no problem), score of 8–10 (possible clinical problems), and score of 11 or higher (evident clinical problems). Responders with HADS-A/D scores of 10 or less (non-anxiety group, non-depression group) and those with HADS-A/D scores of 11 or higher (anxiety group, depression group) were compared. Chronic pain was defined as pain experienced at least once in the past 30 days, with severity of 5 or more on a visual analogue scale (VAS), and persisting for 6 months or more, similar to the definition adopted in the 2010 and 2011 surveys [2, 3]. Furthermore, the age, gender, treatment period, frequency of change of the treating facility, VAS score, PainDETECT score, HADS score and PCS score in the responders with persistent chronic pain were compared among medical facilities and folk remedies. For inter-group comparison, *t* test or ANOVA was used for continuous variables and the Chi-square test or Fisher’s exact test for categorical variables. This study was approved by the IRB of Keio University.

## Results

### Characteristics of the responders complaining of persistent chronic pain

**Fig. 1:**
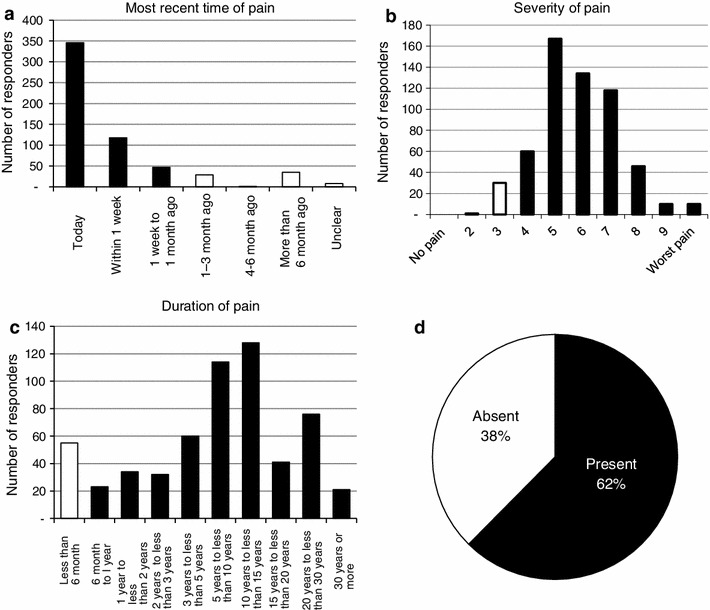
**a** Most recent time of pain, **b** severity of pain (visual analog scale), **c** duration of pain, **d** prevalence of chronic musculoskeletal pain

**Fig. 2:**
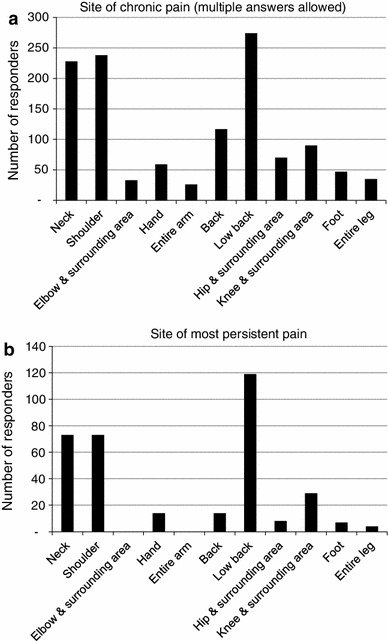
**a** Site of chronic pain (multiple answers allowed), **b** site of the most persistent pain

According to the definition of chronic pain, 365 (62 %) of the 588 respondents had persistent chronic pain, while the remaining 223 respondents (38 %) no longer complained of chronic pain. A noteworthy finding was that the most frequently recorded duration of pain was 10–15 years, and the second most frequently recorded duration was 5–10 years (Fig. 1). The most frequently recorded site of pain was the low back (75 %), followed by the neck and shoulder (about 60 %), similar to the results of the previous surveys (Fig. 2a). When individual respondents were questioned about the site of the most persistent pain, the most frequent response was the lower back (33 %), followed by the neck and shoulder (Fig. 2b).

### Treatment status among responders complaining of persistent chronic pain

**Fig. 3:**
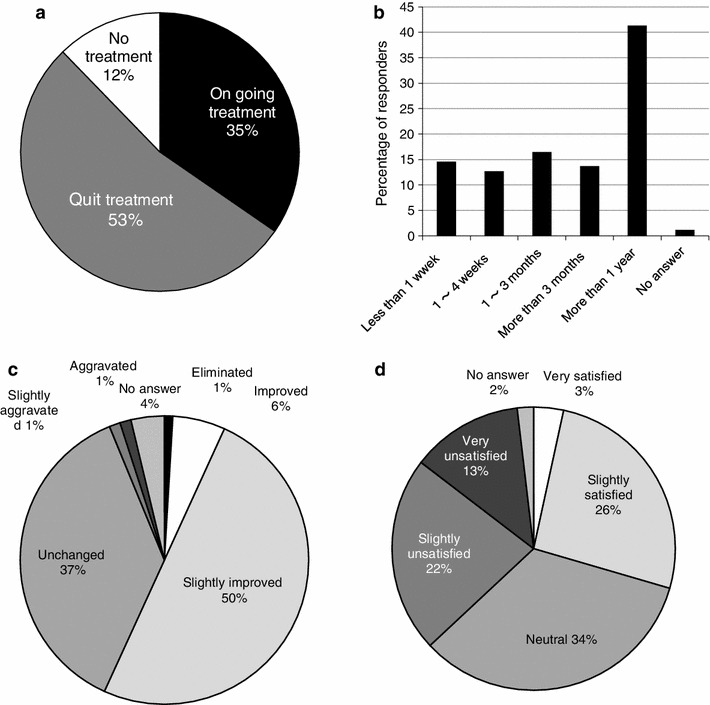
Treatments received for persistent, chronic pain: **a** treatment circumstances, **b** duration of treatment, **c** efficacy of first treatment, **d** degree of satisfaction with first treatment

Of the 365 responders complaining of persistent chronic pain, 128 (35 %) were still receiving treatment at the time of the survey, while 193 (53 %) had discontinued treatment. Forty-four responders (12 %) were not receiving treatment despite the presence of persistent pain (Fig. 3a). The treatment period was 1 year or longer in about 40 % of all respondents, indicating a tendency for prolonged treatment (Fig. 3b). When questioned about the outcome of treatment at the first treating facility, the responses were “disappeared, improved or slightly improved” in 57 %, and “unchanged, slightly aggravated or aggravated” in 39 % (Fig. 3c). The degree of satisfaction with treatment was “very satisfied or slightly satisfied” in only 29 %, and “neutral, slightly unsatisfied or very unsatisfied” in as many as 69 % of the cases (Fig. 3d).

**Fig. 4:**
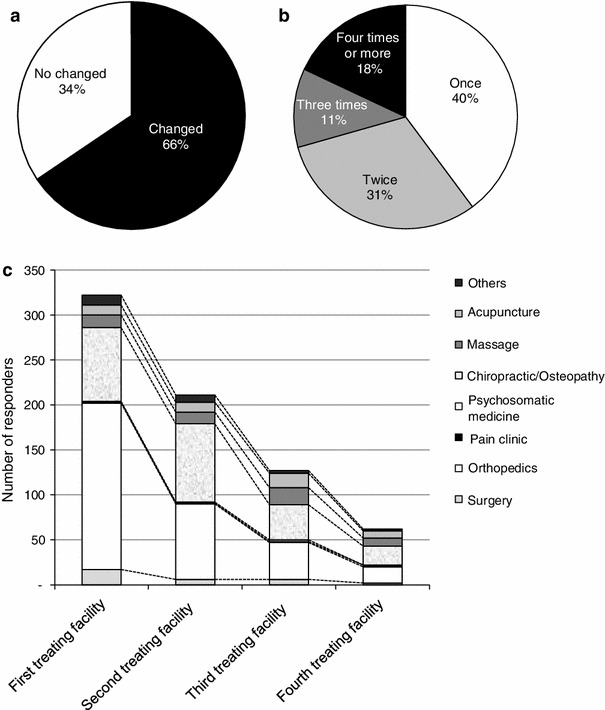
Circumstances of changes in treatment facility: **a** whether changed, **b** frequency of change, **c** history of change of the treatment facility

As a result, the responders often changed the treating facility (66 %), with the frequency of change being once in 40 %, twice in 3 %, three times in 11 %, and 4 times or more in 18 % of the cases (Fig. 4a, b). In a further analysis of the changes in the treating facility, the type of facility providing the initial treatment was most frequently orthopedics (185 responders, 58 %), followed in frequency by a chiropractic/osteopathy (82 responders, 26 %). However, when asked about the type of facility visited as the second treating facility, a smaller number of responders answered “orthopedics” (84 responders) and a larger number of responders answered “chiropractic/osteopathy” (87 responders), with scarce change in the number of responders answering “massage/acupuncture.” When asked about the type of facility visited as the third and subsequent treating facility, the number of responses for each type of facility decreased to a similar degree (Fig. 4c).

**Fig. 5:**
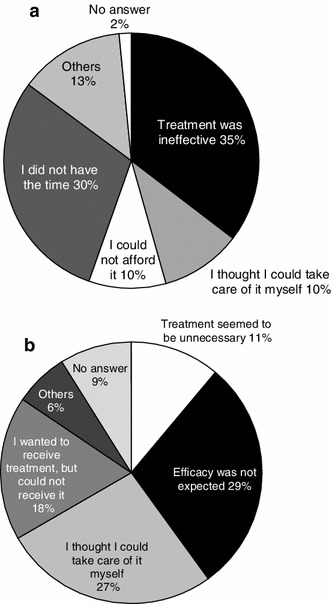
**a** Reason for discontinuation of treatment, **b** reason for seeking no treatment

The most frequent reason for changing the treating facility was “treatment was ineffective” (35 %), followed by “I did not have sufficient time” (30 %), “I thought I could take care of it myself” (10 %), and “it was economically unaffordable” (10 %) (Fig. 5a). The reason for not receiving any treatment was “efficacy was not expected” (29 %), “I thought it may be possible to deal with the pain by myself” (27 %), “I wanted to receive treatment, but could not receive it” (18 %), and “treatment seemed to be unnecessary” (11 %) (Fig. 5b).

### Involvement of neuropathic pain in persistent chronic pain

**Fig. 6:**
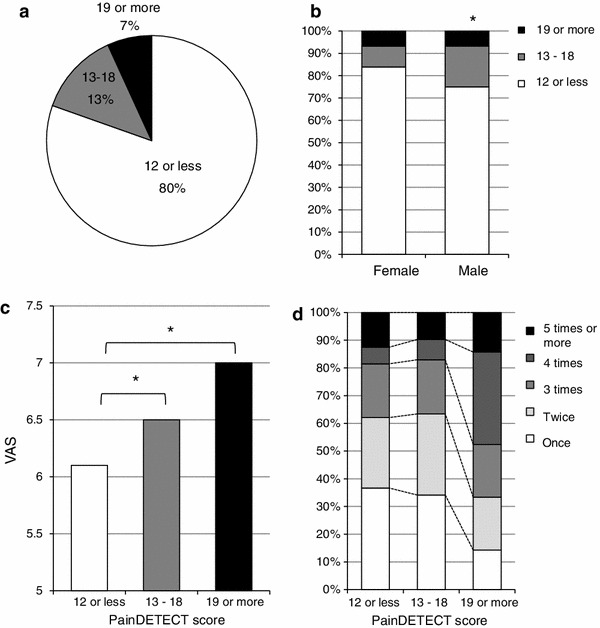
Influence of neuropathic pain on chronic pain: **a** distribution of the painDETECT scores, **b** comparison of painDETECT scores between males and females (*p = 0.06), **c** correlation between painDETECT scores and VAS scores (*p < 0.05), **d** influence of painDETECT score on frequency of change of the treatment facility

Involvement of neuropathic pain in the responders complaining of persistent chronic pain was investigated through analysis of the PainDETECT scores. The percentage of responders classified into the NP group was 7 %, and that of responders classified into the Suspect NP group was 13 % (Fig. 6a). In an analysis of the relation to gender, involvement of neuropathic pain was seen more frequently in males than in females with a marginal significance (*p* = 0.06) (Fig. 6b). In the analysis of the relationship between the VAS score and PainDETECT score, the VAS scores differed significantly among the three groups divided according to the involvement of neuropathic pain. There were significant differences in VAS scores between the non-NP and Suspect-NP groups (*p* = 0.043), and between the non-NP and NP groups (*p* < 0.001, Bonferroni post-hoc test) (Fig. 6c). There was a significant difference in the frequency of change of the treating facility among the three groups (*p* < 0.05). Even after removing the influence of VAS score, covariance analysis revealed that the frequency of change of the treating facility was lower in the non-NP group compared to the NP group with a marginal significance (*p* = 0.056) (Fig. 6d).

### Involvement of psychogenic pain in persistent chronic pain

**Fig. 7:**
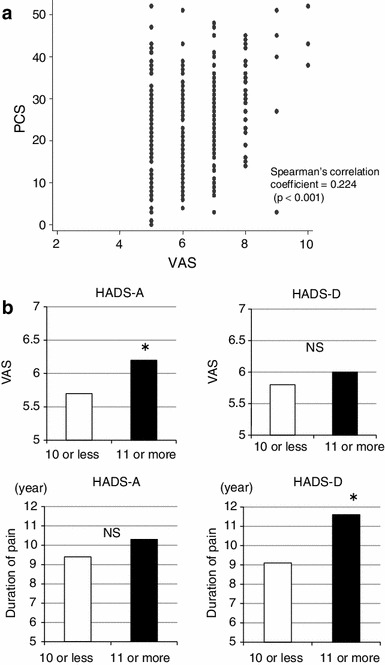
Influence of psychogenic pain on chronic pain: **a** correlation between PCS and VAS scores, **b** influence of HADS-A (anxiety) and HADS-D (depression) scores on the VAS score and duration of pain (*p < 0.05)

The involvement of psychological factors in chronic musculoskeletal pain was investigated through analysis of the correlation between the VAS scores and PCS scores. This analysis revealed a weak but statistically significant positive correlation between the VAS and PCS scores (Spearman’s correlation coefficient = 0.224, *p* < 0.001) (Fig. 7a). When analyzed in relation to the HADS-A score, the VAS score was significantly higher in the responders classified into the anxiety group than in the responders classified into the non-anxiety group, while the duration of pain did not differ significantly between the two groups. When analyzed in relation to the HAD-D score, the duration of pain was significantly longer in the depression group than in the non-depression group (*p* = 0.019, covariance analysis with VAS score), while the VAS score did not differ significantly between the two groups (Fig. 7b).

### Characteristics of the responders complaining of persistent chronic pain, analyzed by the type of the first treating facility

The characteristics of the responders complaining of persistent chronic pain were compared between the two groups divided by the type of the first treating facility, i.e., the group which received the first treatment at a medical facility (medical facility group) and the group which received the first treatment at a folk remedy (folk remedy group). The male-to-female ratio did not differ significantly between the medical facility group and the folk remedy group, however, the age of the responders was significantly higher in the medical facility group than in the folk remedy group. There was no significant difference in terms of the treatment period or the frequency of change of the treating facility between the two groups. The VAS score did not differ between the two groups either. The PainDETECT score tended to be higher in the medical facility group (8.3) than in the folk remedy group (6.4), although the difference was not statistically significant (*p* = 0.06). The PCS score was significantly higher in the medical facility group (26.5) than in the folk remedy group (23.2) (*p* < 0.01, Table 1).

**Table 1:** Characteristics of the responders with chronic pain, analyzed by the type of the first treating facility

		Medical facility (*n* = 213)	Folk remedy (*n* = 108)	*p* value*
Sex				
Female	Number (column %)	129 (60.3)	75 (69.4)	0.11
Male		85 (39.7)	33 (30.6)	
Age	Average (SD)	54.8 (14.8)	46.2 (13.8)	<0.01
Duration of treatment (years)	Average (SD)	10.3(9.0)	10.4 (7.2)	0.91
Frequency of change in the treatment facility	Number (column %)			
1		77 (36.0)	34 (31.5)	0.65
2		51 (23.8)	33 (30.6)	
3		43 (20.1)	22 (20.4)	
4		18 ( 8.4)	6 (5.6)	
5 or more		25 (11.7)	13 (12.0)	
VAS	Average (SD)	6.1 (1.1)	6.4 (1.1)	0.13
PainDETECT score	Average (SD)	8.3 (6.7)	6.8 (5.9)	0.06
PainDETECT				
12 or less	Number (column %)	146 (76.4)	86 (83.5)	0.34
13–18		29 (15.2)	12 (11.7)	
19 or more		16 ( 8.4)	5 (4.9)	
PCS score	Average (SD)	26.5 (10.3)	23.2 (9.9)	<0.01

* *t* test, χ^2^ test, Fisher’s exact

## Discussion

### Current status of responders complaining of persistent chronic musculoskeletal pain

Of the responders who complained of chronic musculoskeletal pain at the time of the survey in 2010, 45 % continued to complain of chronic pain in the 2011 survey, and the percentage of responders still complaining of chronic pain rose to 62 % in the survey of 2012. This result suggests that relief from chronic musculoskeletal pain becomes more difficult as the duration of chronic pain increases. In the present survey, the mean VAS score was higher than the score recorded in the 2010 survey, and the most frequent site of pain was the low back (70 %), suggesting the possibility that many of the responders complaining of chronic pain in this survey had intractable low back pain. This finding is consistent with the results of the longitudinal epidemiological survey of 2011, in which the pain in the “low back” as the site of pain and pain for “5 years or longer” as the duration of pain were suggested as risk factors for the persistence of chronic pain [3]. Past reports have also suggested that lower back pain is associated with a high risk of relapse and a chronic course [7–11]. Therefore, approaches for dealing with the high-risk group will become more important when countermeasures against chronic musculoskeletal pain are discussed.

### Problems with treatment of persistent chronic pain and countermeasures

Slightly more than 80 % of all responders complaining of persistent chronic pain had a history of treatment, with the treatment still continuing in 30 % of the respondents at the time of the present survey. The remaining 50 % were no longer receiving treatment despite persistent pain. When asked about the efficacy of treatment, about 40 % answered “unchanged” or “aggravated,” with the degree of satisfaction with the treatment being “neutral,” “slightly unsatisfied” or “quite unsatisfied” in about 70 %. This tendency was similar to that seen in the 2011 survey. Thus, 66 % of respondents complaining of chronic pain changed the treating facility, with the frequency of change being once or twice in about 70 % of the responders who changed the treating facility. To our surprise, 30 % of the responders changed their treating facility three or more times, suggesting that the percentage of responders engaging in so-called “doctor shopping” cannot be ignored. When changes in the type of the treating facility were analyzed, the first treating facility was an orthopedics in slightly more than 60 % of all responders, while the share of orthopedics as the treating facility decreased to about 50 % after the first change of the treating facility. There was, however, no marked change in the share of folk remedies as the treating facility. This result is consistent with the finding from the survey of 2011, which revealed that the degree of satisfaction with treatment at medical facilities was lower than that at folk remedies [3], suggesting that the initial treatment provided at medical facilities may not be adequate. However, there was no marked difference between medical facilities and folk remedies in terms of the tendency towards subsequent changes of the treating facility. The most frequent reason for changing the treating facility or discontinuing treatment was “treatment was ineffective,” indicating that the current approach for treating chronic musculoskeletal pain may not be sufficiently effective. To identify the factors possibly underlying this finding, we investigated the involvement of neuropathic pain and psychogenic pain in persistent chronic musculoskeletal pain.

This analysis suggested possible involvement of neuropathic pain in about 20 % of all responders complaining of chronic pain. It was additionally revealed that the VAS score rose significantly and the frequency of change of the treating facility also increased as the likelihood of involvement of neuropathic pain became higher. Regarding psychogenic pain, a significant positive correlation was noted between PCS and VAS scores, an increase in HADS-A score was associated with an increase of the VAS score, and an increase in the HADS-D score was associated with a longer duration of pain. In regard to chronic low back pain, which was the most frequent type of pain recorded in the present survey, the previously reported important role of psychogenic factors [12–16] was also endorsed by the results of the present survey. Interestingly enough, analysis of the characteristics of the responders complaining of chronic pain in relation to the type of the first treating facility revealed that medical facilities more frequently managed patients of advanced age and with a stronger likelihood of involvement of neuropathic pain and psychogenic pain than folk remedies. These factors may explain, at least partially, the relatively low satisfaction level of responders with the treatment at medical facilities. However, caution is needed while interpreting the results as to psychogenic pain, in view of the possibility that treatment may result in progression of catastrophic thinking or depressive mood.

Taken together, these results suggest that many of the patients complaining of chronic musculoskeletal pain seek treatment at the orthopedic clinic/department first, but tend to show low levels of satisfaction with the treatment because of insufficient efficacy, and that neuropathic pain and psychogenic pain may be involved in the poor responses of these patients to treatment. Lack of adequate assessment for neuropathic and psychogenic pain during the initial treatment of chronic musculoskeletal pain and the resultant absence of appropriate treatment seem to lead to “doctor shopping” by patients. A past report also pointed out the close involvement of neuropathic pain with chronic low back pain [17]. To resolve this issue, it will be important to assess the involvement of neuropathic pain on the basis of the PainDETECT score and neuropathic severity score before treatment is started in individual patients complaining of chronic musculoskeletal pain. Furthermore, the results of the present survey suggest that if treatment is provided in a manner tailored to the status of involvement of psychogenic pain rated by the HADS and PCS, it may become possible to reduce the intensity of pain and shorten the duration of pain.

Many previous reports have shown that chronic musculoskeletal pain can impair not only physical health, but also mental health, which may have a large impact on the daily living and social activities of the patients [2, 18]. However, the awareness among patients about chronic pain does not seem to be sufficient, considering the finding from this survey that patients often decided to discontinue treatment or seek no treatment for chronic pain persisting for 3 years or more, on grounds such as “I did not have sufficient time,” “I thought I could take care of it myself” and “I thought treatment was unnecessary.” At present, the actual status of chronic musculoskeletal pain is not sufficiently well understood by the people in Japan. Dissemination of information through various media to deepen the understanding of the people is important for ensuring a sufficient level of awareness among the people of Japan about the significance of chronic musculoskeletal pain treatment.

This study was conducted as a 2011 Ministry of Health, Labour and Welfare: Health Labour Sciences Research Grant for Comprehensive Research on Disability Health and Welfare (Survey study of chronic musculoskeletal pain).
